# Complementary and Alternative Medicine Use in Hospitalized Cancer Patients—Study from Silesia, Poland

**DOI:** 10.3390/ijerph19031600

**Published:** 2022-01-30

**Authors:** Karolina Kasprzycka, Marta Kurzawa, Malgorzata Kucharz, Monika Godawska, Marta Oleksa, Marta Stawowy, Katarzyna Slupinska-Borowka, Wiktoria Sznek, Iwona Gisterek, Agnieszka Boratyn-Nowicka, Monika Rucinska, Karolina Osowiecka, Sergiusz Nawrocki

**Affiliations:** 1 Department of Oncology and Radiotherapy and Affiliated Student Science Club, Medical University of Silesia in Katowice, ul. Ceglana 35, 40-514 Katowice, Poland; k.kasprzycka@gazeta.pl (K.K.); mrtkurzawa@gmail.com (M.K.); gkucharz@gmail.com (M.K.); monikagodawska@hotmail.com (M.G.); marta.oleksa@gmail.com (M.O.); martastawowy@gmail.com (M.S.); katarzynajuliaes@gmail.com (K.S.-B.); wsznek@gmail.com (W.S.); igisterek@sum.edu.pl (I.G.); sergiusz.nawrocki@me.com (S.N.); 2 Katowice School of Technology, University of Technology in Katowice, ul. Rolna 43, 40-555 Katowice, Poland; aboratynnowicka@zco-dg.pl; 3 Department of Oncology, Collegium Medicum, University of Warmia and Mazury in Olsztyn, 10-228 Olsztyn, Poland; m_rucinska@poczta.onet.pl; 4 Department of Psychology and Sociology of Health and Public Health, School of Public Health, University of Warmia and Mazury in Olsztyn, 11-041 Olsztyn, Poland

**Keywords:** CAM, complementary medicine, alternative medicine, cancer patients

## Abstract

Background: The use of complementary and alternative medicine (CAM) is common amongst cancer patients. The aim of the study was to investigate the use of CAM, beliefs about CAM and the purpose of using it amongst Polish cancer patients. Methods: The study included 864 cancer patients (median 63 years old), who were individually interviewed. The questionnaire was designed specifically for this study. Results: Amongst 732 patients who declared that they heard about CAM, 342 patients (46.7%) had used CAM; 91% of these patients had used it as a complementary therapy and 9% had used it as an alternative therapy. Patients younger in age, highly educated, professionally active, with longer medical history, and at more advanced cancer stages have, statistically, used CAM more often; 66% of participants could not state what the health effect of CAM is. Patients received information about CAM from the internet, friends, family and other patients. Only 18% of patients discussed using CAM with a doctor. Conclusions: CAM was popular amongst Polish cancer patients, especially in younger, educated and professionally active patients with longer cancer history at advanced stage. Patients used CAM as a complementary therapy for strengthening immune system, improving morphological and biochemical test parameters, reducing the side effects of conventional therapy and improving their well-being.

## 1. Introduction

Some patients use health care approaches that are not typically a part of conventional medical care—complementary and alternative medicine (CAM). CAM includes a wide range of products and medical practices that are not a conventional therapy in Western countries. According to the National Center for Complementary and Integrative Health (NCCIH), complementary medicine is not the same as alternative medicine. If some unconventional approach—action or product—is used together with conventional medicine, it is called complementary. If an unconventional approach is used in the place of standard treatment, it is considered alternative. CAM therapies include herbs, vitamins, minerals, probiotics, nutritional supplements and special diets. Practices such as deep breathing exercises, yoga, tai chi, massage, chiropractic manipulations, magneto-therapy and acupuncture also belong to CAM. Psychological CAM include relaxation techniques, qi gong, hypnotherapy, meditation, music/dance therapy and mindfulness-based stress reduction. In Western medicine in recent years, a new approach has appeared—integrative health. In an integrative health approach, CAM is used together with conventional medicine in a coordinated way. Some extra actions, for example lifestyle changes, rehabilitation, psychotherapy and complementary health approaches, can be beneficial during conventional anti-cancer treatment. It is important to provide this kind of care in a coordinated way and to adapt it individually to a given patient’s needs [1].

The use of unconventional products and activities is common amongst both healthy and sick populations. Around 36% of cancer patients in Europe use CAM and the number of patients using unconventional therapies is growing [2]. Cancer patients in particular are willing to explore to alternative therapies. The prevalence of CAM use amongst cancer patients is in the range of 9–88% and depends on country and cancer type [3]. Meta-analysis of patient data found an increase in CAM use by cancer patients from 25% in the 1980s to 32% in the 1990s and 49% in the 2000s [3]. Delay or refusal of conventional cancer treatment, due to a belief in alternative medicine, may have a negative impact on survival for cancer patients [4,5,6,7,8,9,10]. In Poland, there is still a lack of studies about CAM use among cancer patients. In 2017, there was an intensive discussion in the popular press; the two most widely read Polish magazines were alarmed, reporting that about 75% of cancer patients used some unconventional therapies [11,12], and that some patients even resigned from conventional, evidence-based treatments. However, it was only a speculation, which was not based on systematic studies or solid data, and was mostly based on interviews with expert oncologists and individual patients. Therefore, at that time, we decided to carry out this study. The aims of this study were to investigate CAM popularity among cancer patients in Poland, explore patients’ insights and beliefs about CAM, and evaluate their goals and expectations when using complementary and alternative medicine.

## 2. Materials and Methods

The study was carried out between 15 January 2018 and 28 March 2019 in 4 oncological centers in the southern region (Silesia) of Poland. A total of 864 hospitalized patients treated for malignant neoplasm took part in our study. The study was planned and carried out by final-year medical students studying oncology. The students worked under the supervision of oncologists and biostatisticians. A questionnaire was designed specifically for this study, in accordance with approved principles [13,14]. The questionnaire consisted of 24 main closed questions, with some questions allowing further options for respondents to complete when answering: 15 questions allowed the patient to select a single response, 7 questions allowed the patient to select 1 or more responses (as applicable to them) and 2 questions provided the patient with a scale of responses to select from (see Supplementary Files S1). Demographic data included age, gender, education, profession, working status, place of residence and some information about cancer diagnosis (tumor localization, date of diagnosis, stage of disease, intention of treatment). Patients were individually interviewed by students during the research survey for more reliable data collection. Students supported patients in reading and in their understanding of the questions and difficult phrases, encouraging them to give honest answers.

**Figure 1:**
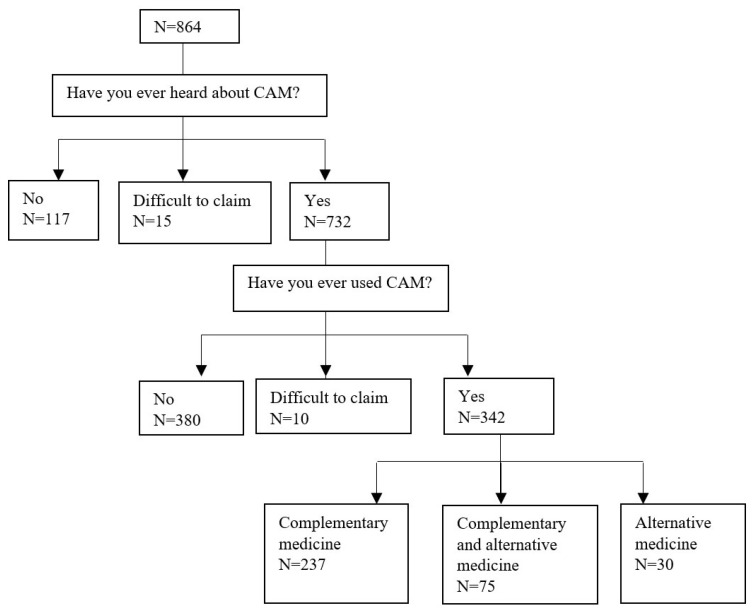
Flow diagram of patients included in the study.

If patient had never heard about complementary and alternative medicine (CAM), they were excluded from further analysis. A total of 132 patients (15%) of the 864 interviewed were excluded (117 patients were unaware of CAM and 15 patients were unable to provide a definitive response). Amongst the 732 patients who had indicated that they had knowledge of CAM, 342 patients (46.7%) declared use of CAM. There were 10 patients (1.4%) who were unable to provide a definitive response, and they were excluded from the comparative analysis. Of the 342 participants who used CAM, 237 patients (69%) declared use of complementary medicine, 75 patients (22%) declared use of complementary and alternative medicine, and 30 patients (9%) declared use of alternative medicine (Figure 1).

The comprehensibility and acceptability of the questionnaire was validated by the physician (specialized in oncology) and the psycho-oncologist. A pilot study was conducted on a group of 20 patients, and the questionnaire was modified based on the results from the pilot. The reviewers verified that the questions were appropriately clear and unambiguous, based on answers from the first 20 patients. In a group of 38 patients, the reliability was then estimated. The validation procedure included a questionnaire that was carried out twice in a two-week interval on the same group of patients. An estimation of the degree of reproducibility of individual responses was made using Cohen’s kappa coefficient of agreement. The repeatability was good (29%) and very good (71%), respectively.

The study protocol was presented to the Local Ethics Committee of the Medical University of Silesia in Katowice, Poland. The Local Ethics Committee decided that approval for this kind of study is not required. By agreeing to fill in the questionnaire, the patients gave their consent to participate in the study.

### Statistical Analysis

Descriptive statistics were used (median, average, standard deviation, percentages). The chi-square test was used to compare the response prevalence between CAM users vs. non-users, associated with various factors. The age and time since diagnosis differences for each question were estimated using the Mann–Whitney test. The odds ratio (OR) with 95% confidence interval (CI) of using CAM was estimated in univariate and multivariate analysis using the logistic regression model. The variables with *p* ≤ 0.1 in univariate analysis were included in multivariate model. A *p* value < 0.05 was considered to be significant. The analysis was conducted using Statistica (data analysis software system), version 13 (http://statistica.io (accessed on 1 January 2018)) TIBCO Software Inc., Krakow, Poland (2017).

## 3. Results

The study was carried out on a group of 864 cancer patients. A total of 732 patients, who had heard about CAM, were included to the analysis. Patients were in age range 18–93 years (median 62 years old). There were 407 women (55.6%) and 325 men (44.4%); 66.9% of patients had secondary- or higher-level education. More than half of patients were retired (58%). Over 40% of respondents lived in cities of 50,000–200,000 inhabitants, 22% of respondents lived in big cities (>200,000 inhabitants) and 15% of respondents resided in villages. The most frequent diagnosis for study participants was colon cancer (23%), followed by upper digestive system cancer (16%), lung cancer (12%) and breast cancer (12%). Half of patients were treated with the curative intent. A quarter of patients (26.5%) were being treated for clinical stage IV disease (metastatic disease). The median time since cancer diagnosis was approximately 0.6 year (Table 1).

A total of 342 patients declared that they had used complementary and/or alternative medicine. Patients who were younger, highly educated and professionally active, with a longer period since diagnosis and at more advanced cancer stage had, statistically, more often used CAM. In the univariate analysis age, place of residence and stage of disease were significantly correlated with OR of CAM usage. In the multivariate model, the more advanced stage of cancer was noted as an independent factor increasing the probability of CAM usage in patients (Table 1).

Amongst 732 patients who had knowledge of CAM, 18% had engaged in a conversation with a doctor about CAM. Most participants (66%) had not experienced any health benefits of CAM; 18% of participants indicated that, in their opinion, CAM had harmful effects on health. Over a quarter of participants (28.7%) claimed that CAM, used with cancer treatment, should be regulated by law.

**Figure 2:**
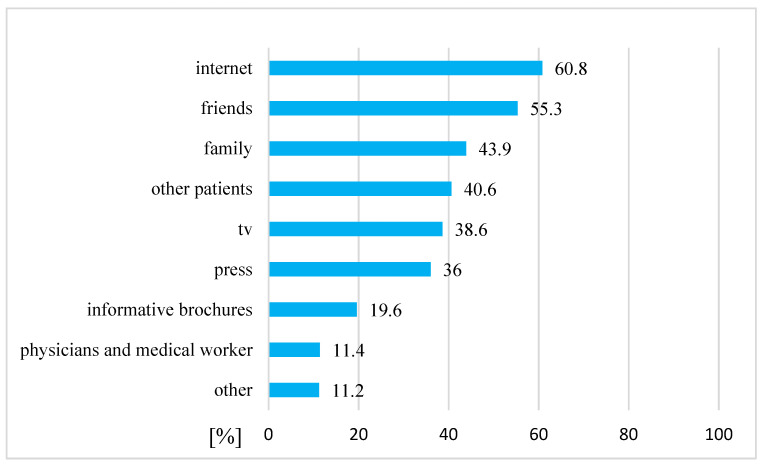
The sources of information about CAM amongst cancer patients who had used complementary and/or alternative medicine (n = 342).

Amongst 342 cancer patients who declared that they had used complementary and/or alternative medicine, the information they had about CAM was mostly obtained from the internet, friends, family and other patients (Figure 2).

**Figure 3:**
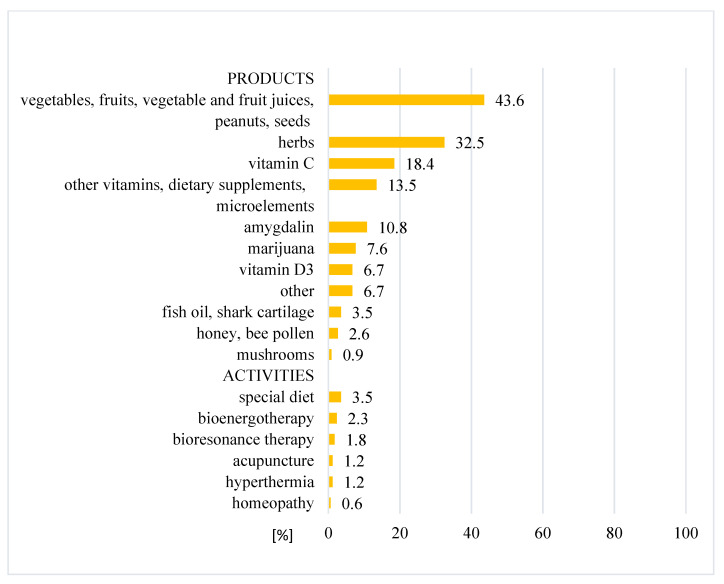
Distribution of used CAM modalities.

The CAM treatments that were used the most by patients in this study were natural products (43.6%), herbs (32.5%), vitamin C (18.4%) and other vitamins, dietary supplements and microelements (13.5%). Some activities, such as special diets, bioenergy therapy, bioresonance therapy, hyperthermia or acupuncture, were used less frequently (1–3.5%). Patients, who declared that they had used alternative medicine had mainly used high doses of vitamin C, amygdalin, herbs and marijuana (Figure 3).

**Figure 4:**
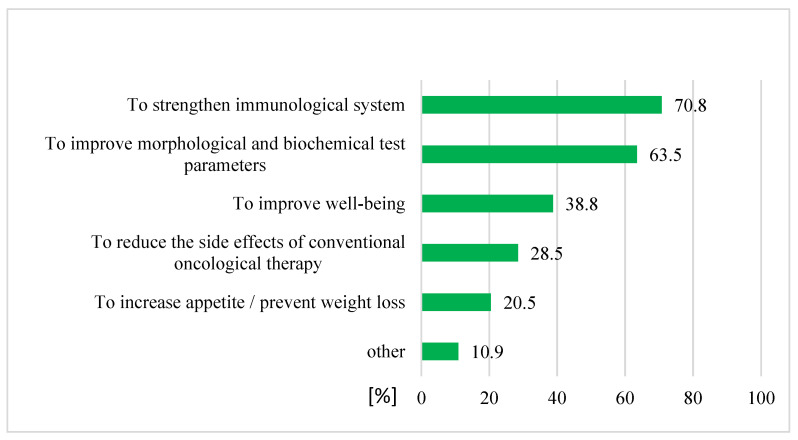
Reasons for CAM use as a complementary medicine (n = 312).

**Figure 5:**
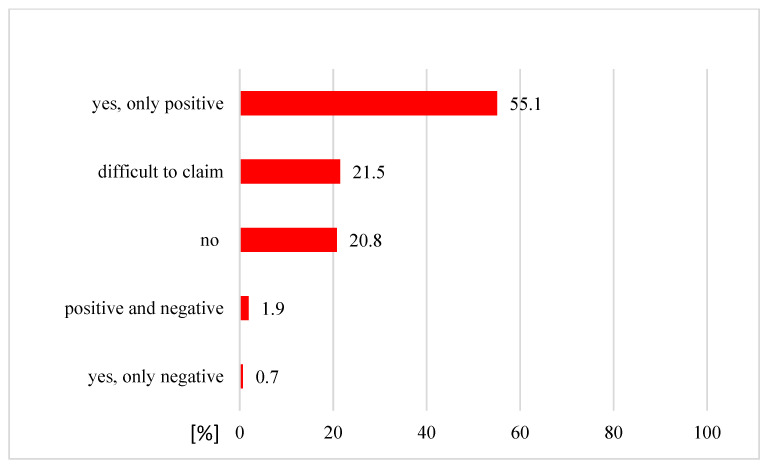
Perceived effects of CAM as a complementary medicine (n = 312).

Of the 342 patients who claimed that they had used CAM, 312 patients (91%) had used it as a complementary medicine and 30 patients (9%) undertook CAM as an alternative medicine. The use of complementary methods was mostly undertaken with the aim of strengthening the patient’s immune system (70.8%) and in the interest of improving morphological and biochemical test parameters (63.5%) (Figure 4). More than half of patients who had used a complementary medicine (172 patients, 55%) had noticed the positive effects of CAM therapy (Figure 5).

All patients, except 2, who stated that they were using CAM as an alternative therapy (28 patients) used it together with traditional cancer treatment. Only one patient claimed they were using alternative therapy instead of conventional therapy, and one patient used CAM after completion of their conventional treatment; additionally, 60% of patients did not inform their oncologist about their use of CAM. Half of the patients interviewed expected that CAM would increase the effectiveness of conventional cancer treatment, but positive effects of unconventional methods were only reported by 16.7% of patients in this group, and half of patients stopped using CAM. The most common reasons for discontinuation of CAM were no positive effects (23.3%) and running out funds to continue the therapy (16.7%); 30% of patients using CAM as an alternative method would not recommend it to other patients (Table 2).

## 4. Discussion

### 4.1. Prevalence and Reasons for CAM Use

Cancer diagnosis is a very stressful situation. Oncological treatment takes a long time, causes a lot of side effects and decreases patients’ quality of life. Some patients decided to use CAM because they wanted to decrease the side effects which they were experiencing from conventional therapy and wanted to improve their well-being. Other patients sought complementary and alternative methods of treatment with the hope that unconventional therapies would be more acceptable and comfortable for them. The overall prevalence of complementary and alternative medicine use by cancer patients is diverse between countries; 36% of cancer patients in European countries use CAM [15], and the percentage of cancer patients treated with CAM is 46% in Japan [16], 60% in Canada [17], 70–83% in the US [18,19], 85% in Tunisia [20] and 98% in Shanghai [21].

In our study, 13.5% of patients were not familiar with CAM. Half of patients (47%) who had heard about CAM decided to use it; however, most of them (91%) used CAM as a complementary therapy, rather than as the primary treatment for cancer. In Saudi Arabia, most patients (75%) reported using CAM to treat cancer in [22]; additionally, 44.5% of Japanese patients using CAM claimed that they used complementary and alternative medicine to cure cancer [16]. Buckner et al. [17] reported that 51.6% of Canadian patients using CAM declared that they were “just trying to do everything that can help”. Other reasons for CAM use included strengthening the immune system and in attempts to improving sleep and relaxation. In France, most patients indicated that their reason for using CAM was to prevent or treat the side effects of conventional therapy (81%), and about half of patients reported that their reason was to improve their well-being [23]. Half of Italian cancer patients claimed that they used CAM, and of these, most of them thought that CAM could decrease the side effects of chemotherapy [24]. Another Polish study demonstrated that CAM is commonly used by patients following a primary diagnosis of cancer. Half of patients (49%) hoped to enhance the effect of conventional therapy, whilst 16% hoped to treat the side effects [25]. The main reasons that patients in our study used CAM were the following: strengthening the immune system (71%), improvement of morphological and biochemical test parameters (64%), improvement of well-being (39%), and reducing the side effects of conventional therapy (29%). Similar results were reported amongst cancer patients in Sweden [26].

In our study, only 1 patient out of 342 patients using CAM decided to resign completely from conventional treatment. In Saudi Arabia, where a large proportion of patients use CAM and believe that CAM could cure cancer, only about 3% delayed beginning their conventional treatment in favor of CAM therapy [22]. In the analysis of Tascilar et al. [27], most patients from the countries studied used CAM together with conventional treatments.

### 4.2. Predictors of CAM Usage

In our study, patients of a younger age, who were highly educated and professionally active, with a longer period since diagnosis and at more advanced cancer stages, were more likely to use CAM. More advanced stage of disease was an independent factor for CAM use by cancer patients. Being female, of a younger age and highly educated predicted CAM use in other populations [16,20,22,24,25,26,27,28,29,30,31]. Additionally, CAM users were more likely to have advanced stages of cancer (IV) and have a history of delayed diagnosis [32]. CAM were more likely to be used by breast cancer and lung cancer patients [24]. In contrast, in our study, there were no significant differences among the prevalence of CAM usage based on cancer types.

### 4.3. Types of CAM

The types of CAM most frequently used by patients in our study were natural products, herbs, vitamin C and other vitamins, dietary supplements and microelements. The same therapies have commonly been reported by other authors [17,24,25,26,28,29,30,31,32,33]; however, there are some therapy preferences that have been reported to be nation-specific. In the USA, the most popular CAM were vitamins, minerals, herbal supplements and green tea [28,29]. In Sweden, the most popular were vitamins, minerals and relaxation [26]. In Japan, patients often used mushrooms, propolis and Chinese herbs. In Saudi Arabia, some of the most commonly used CAM treatments were found to be Zamzam water, camel milk and olive oil [16,22]. People in Saudi Arabia also considered supplication and Quran recitation as part of CAM [22]. Americans often engaged in spiritual practices—meditation and prayer [29,30].

### 4.4. Sources of Information about CAM

Patients often do not inform their doctors that they are using CAM; however, doctors do not ask their patients whether they are using additional therapies. In our study, only 18% of patients who were aware of CAM talked with their physicians about the possibility of using these methods in their treatment. Most of the patients (60%) who reported using anti-cancer CAM did not inform their oncologist. Results from other studies confirmed that most of patients did not inform their oncologist about CAM [20,26]. Hyodo et al. [16] reported that over 60% of patients did not consult with a doctor over their usage of CAM. In France, only 33.5% of cancer patients using CAM discussed it with oncologists [23]. However, in Italy, most CAM users informed their oncologists [24]. Patients declared that they did not talk about CAM with doctors for various reasons, including fear of disapproval, exclusion from clinical trials or simply because no health care professionals asked them [34,35]. In our study, patients mostly obtained information about complementary and alternative medicine from the internet, friends, family and other patients. In various other studies, the same sources of information on CAM were the most popular amongst cancer patients [17,20,22,24,33,36]. The Pew Internet Project for Health reported that more than 60% of people who used the internet were looking for health-related information, half of whom were looking for information on CAM [37]. Only about 6% of Italian cancer patients had heard about CAM from a doctor [24]. Clinicians often do not ask patients about CAM use [38,39]. It could be a good idea to research whether doctors and other medical professionals (e.g., nurses) are interested in their patients’ CAM use in future studies.

### 4.5. Experiences and Recommendation of CAM Use

Our study showed that more than half of patients (55%) reported positive effects of complementary and alternative medicine. Amongst patients who used CAM as an anti-cancer therapy, only 16.7% reported positive effects (in their experience, CAM improved their mental health and well-being, physical condition, reduced the side effects of conventional therapy or had an anti-cancer effect). Half of patients using CAM as an anti-cancer therapy (50%) discontinued using CAM because of no positive effects; 30% of patients using complementary and alternative medicine as an anti-cancer treatment would not recommend it to other patients. In Sweden, a few CAM users (5.6%) reported adverse effects from CAM modalities [26]. Berretta et al. [24] demonstrated that beneficial effects of using CAM were reported by 34.5% of patients, whereas more than half of them (59%) had no opinion about effects; however, 87.3% of respondents said they would recommend CAM, and 67.2% trusted in CAM. In Japan, 24.3% of cancer patients reported beneficial effects of CAM, such as pain relief, decreasing the side effects of conventional treatment, reducing tumor growth and improved well-being. About 70% of patients did not know whether CAM was effective [16]. Only a small percentage of patients using CAM reported side effects from the therapy [16,24]. In our study, 6.7% of patients using CAM as an alternative therapy reported negative effects, and only 2.6% of patients were using CAM as a complementary treatment. It is difficult to evaluate the adverse effects of overall CAM. Complementary and alternative medicine encompasses a diverse range of therapies and methods. Some CAM, such as acupuncture, meditation, massage and music therapy could be in some way beneficial for cancer patients and could improve their general health [40,41]. However, there are also known adverse interactions between CAM and some anti-cancer drugs (e.g., inhibition or activation of cytochromes) [24,27,42]. A significantly high adverse effect on survival was noted in cases of patients using CAM who delayed their conventional cancer treatment in favor of CAM; the five-year overall survival was significantly worse in cases of breast, lung and colorectal cancer patients [43]. Johnson et al. [44] showed that CAM use was associated with a significantly higher risk of death, because patients resigned from at least one standard oncological method of treatment. We noted that almost one in three of the patients surveyed in the present study believed that CAM should be regulated by law. CAM use by cancer patients is common. The Society of Integrative Oncology recommends integration of evidence-based CAM into regular oncologic care. Guidelines have been prepared based on reliable sources of information to help patients understand the risks and benefits of using CAM [45].

### 4.6. Limitations

Our study has some important limitations. One is the selection of a subpopulation of cancer patients who have been actively diagnosed or treated within oncological hospitals in stationary wards. Our study did not include patients who are not treated in a conventional way (for reasons such as refusal, very advanced disease, comorbidities, etc.) and we also did not include outpatients.

Our study was conducted across several hospitals in a large, highly urbanized and industrialized region; therefore, it may not be representative of the Polish population as a whole.

## 5. Conclusions

Almost half of the cancer patients surveyed in the present study reported using complementary and alternative medicine as a supportive therapy for strengthening their immune system, for improvement of morphological and biochemical test parameters, to reduce the side effects of conventional therapy and/or for the improvement of well-being. CAM use was most prevalent amongst patients of a younger age, who were highly educated and professionally active, with a longer period since diagnosis and at a more advanced cancer stage. Patients obtained information about CAM from the internet and through friends/family, rather than a doctor. Although a lot of cancer patients in Poland were found to use CAM, very few patients replaced or stopped their conventional treatment in favor of alternative medicine. Some alarming news about overuse and misuse of CAM therapies, reported in the popular press, were not confirmed by our study results. The contribution of our study to the health care service may be that Polish cancer patients need a little more education on CAM therapies—patients generally seek information about cancer treatment on the internet; therefore, there should be reliable services for patients, providing evidence-based CAM information.

In terms of cancer patients’ needs, this study shows that a lot of patients used CAM, and seeking extra support was not unusual among patients. The main reasons for CAM use were improvement of blood parameters and quality of life, but most of patients (66%) did not notice any positive effects of CAM. Of interest is that only 40% of patients who had used alternative medicines would recommend them to other patients.

It would be beneficial to encourage both patients and their doctors to talk about CAM (more than 80% of patients did not discuss it with their physician).

## Figures and Tables

**Table 1:** Characteristics of patients and odds ratio (OR) of CAM usage due to various factors.

Variables		All		CAM Users		CAM Non-Users			OR of CAM Usage					
		N = 732	(%)	N = 342	(%)	N = 380	(%)	*p* *	OR	(95% CI)	*p* **	OR	(95% CI)	*p* ***
Age median (IQR)		62 (55–68)		61 (54–67)		63 (56–69)		0.009	0.99	(0.97–1.00)	0.02	0.99	(0.98–1.00)	0.07
Gender														
	Female	407	(55.6)	199	(58.2)	200	(52.6)	0.13	1.00	Reference				
	Male	325	(44.4)	143	(41.8)	180	(47.4)		1.25	(0.93–1.68)	0.13			
Education														
	Primary	48	(6.6)	18	(5.3)	30	(7.9)	0.04	1.00	Reference		1.00	Reference	
	Vocational	194	(26.5)	76	(22.2)	112	(29.5)		1.13	(0.59–2.17)	0.71	1.01	(0.52–1.97)	0.97
	Secondary	304	(41.5)	153	(44.7)	147	(38.7)		1.73	(0.93–3.25)	0.08	1.47	(0.78–2.80)	0.24
	Higher	186	(25.4)	95	(27.8)	91	(23.9)		1.74	(0.91–3.34)	0.10	1.49	(0.76–2.91)	0.25
Place of residence														
	Village	113	(15.4)	61	(17.8)	50	(13.2)	0.17	1.00	Reference		1.00	Reference	
	City < 50,000 inhabitants	145	(19.8)	67	(19.6)	75	(19.7)		0.73	(0.44–1.21)	0.22	0.72	(0.43–1.19)	0.20
	City 50–200,000 inhabitants	310	(42.4)	133	(38.9)	174	(45.8)		0.63	(0.40–0.97)	0.04	0.64	(0.41–1.00)	0.05
	City >200,000 inhabitants	164	(22.4)	81	(23.7)	81	(21.3)		0.82	(0.50–1.33)	0.42	0.84	(0.51–1.38)	0.49
Professional activity														
	Employed	299	(40.9)	164	(48.0)	130	(34.2)	<0.001	1.00	Reference				
	Pensioner	424	(57.9)	175	(51.1)	244	(64.2)		2.52	(0.62–10.28)	0.20			
	Unemployed	9	(1.2)	3	(0.9)	6	(1.6)		1.43	(0.35–5.81)	0.61			
Cancer localization														
	Colon	170	(23.2)	80	(23.4)	90	(23.7)		1.00	(0.37–2.72)	1.00			
	Upper digestive system	113	(15.5)	46	(13.5)	64	(16.8)	0.38	0.81	(0.29–2.25)	0.68			
	Lung	89	(12.2)	49	(14.3)	39	(10.3)		1.41	(0.50–4.00)	0.51			
	Breast	89	(12.2)	49	(14.3)	38	(10.0)		1.45	(0.51–4.11)	0.48			
	Gynecological	64	(8.7)	29	(8.5)	31	(8.2)		1.05	(0.36–3.09)	0.93			
	Head and neck	42	(5.7)	21	(6.1)	21	(5.5)		1.13	(0.36–3.48)	0.84			
	Urinary system—without prostate	37	(5.1)	16	(4.7)	21	(5.5)		0.86	(0.27–2.72)	0.79			
	Brain	31	(4.2)	12	(3.5)	19	(5.0)		0.71	(0.21–2.35)	0.58			
	Prostate	17	(2.3)	8	(2.3)	9	(2.4)		1.00	Reference				
	Other	80	(10.9)	32	(9.4)	48	(12.6)		0.75	(0.26–2.15)	0.59			
Time since diagnosis (years) median (IQR)		0.62 (0.33–1.54)		0.70 (0.41–1.71)		0.53 (0.28–1.44)		0.002	1.00	(0.99–1.00)	0.13			
Intention of planned treatment														
	Curative	370	(50.6)	171	(50.0)	196	(51.6)	0.83	1.00	Reference				
	Palliative	167	(22.8)	81	(23.7)	83	(21.8)		1.12	(0.77–1.62)	0.55			
	Difficult to evaluate	195	(26.6)	90	(26.3)	101	(26.6)		1.02	(0.72–1.45)	0.91			
Stage of disease														
	Early	163	(22.3)	62	(18.1)	101	(26.6)	0.02	1.00	Reference		1.00	Reference	
	Local and/or regional development	238	(32.5)	120	(35.1)	111	(29.2)		1.76	(1.17–2.65)	0.006	1.69	(1.12–2.56)	0.01
	Metastatic	194	(26.5)	101	(29.5)	92	(24.2)		1.79	(1.17–2.73)	0.007	1.69	(1.10–2.60)	0.02
	Difficult to evaluate	137	(18.7)	59	(17.3)	76	(20.0)		1.26	(0.79–2.01)	0.32	1.25	(0.78–2.01)	0.35

IQR—interquartile range; OR—odds ratio; CI—confidence interval; * *p*-value estimated using chi-square test; ** *p*-value estimated using univariate logistic regression model; *** *p*-value estimated using multivariate logistic regression model.

**Table 2:** The answers of patients who declared that they had used CAM as an alternative medicine (n = 30).

		N	(%)
Form of alternative medicine:			
	Used as the only treatment instead of traditional treatment.	1	(3.3)
	Used as a supportive therapy of traditional cancer treatment.	28	(93.4)
	Used as therapy after completion of traditional treatment.	1	(3.3)
Informed oncologist about used alternative medicine:			
	Yes	10	(33.3)
	No	18	(60.0)
	Difficult to claim	2	(6.7)
Expectations of using alternative medicine: *			
	Complete recovery	3	(10.0)
	Increasing effectiveness of conventional cancer treatment	17	(56.7)
	No precise expectations—using every possible treatment	11	(36.7)
	Other	4	(13.3)
Timing of decision about using alternative medicine:			
	Before traditional cancer treatment	11	(36.7)
	During traditional cancer treatment	18	(60.0)
	When traditional cancer treatment was completed or without expected results	0	(0.0)
	Other	1	(3.3)
Perceived positive effects of alternative medicine:			
	Yes	5	(16.7)
	No	17	(56.7)
	Difficult to claim	8	(26.6)
Perceived negative effects of alternative medicine:			
	Yes	2	(6.7)
	No	27	(90.0)
	Difficult to claim	1	(3.3)
Considering withdrawal from conventional cancer treatment to use alternative medicine:			
	Yes	1	(3.3)
	No	28	(93.4)
	Difficult to claim	1	(3.3)
Recommendation of alternative medicine to other patients:			
	Yes	12	(40.0)
	No	9	(30.0)
	Difficult to claim	9	(30.0)
Have you still used alternative methods?			
	Yes	9	(30.0)
	No	15	(50.0)
	Some of them yes, some of them no	6	(20.0)
Reasons for resigning from alternative medicine: *			
	No positive effects	7	(23.3)
	Negative effects and/or complications	2	(6.7)
	Encouraged to resign use by a doctor/other medical professional	1	(3.3)
	Encouraged to resign use by family/friends	0	(0.0)
	Negative information about using method	0	(0.0)
	Running out the funds to continue the therapy	5	(16.7)
	Other	9	(30.0)

*—multiple choice.

## Data Availability

All data is available at the Department of Oncology and Radiotherapy, Medical University of Silesia in Katowice, Poland.

## Supplementary Materials

The following supporting information can be downloaded at: https://www.mdpi.com/article/10.3390/ijerph19031600/s1, Files S1: The questionnaire (English version).
