# Supplementary material for: Complementary and Alternative Medicine Use in Hospitalized Cancer Patients—Study from Silesia, Poland
Source: Int J Environ Res Public Health. 2022 Jan 30;19(3):1600. doi: 10.3390/ijerph19031600 (PMC8835686; doi:10.3390/ijerph19031600)
Supplement: Supplementary file 1 [file ijerph-19-01600-s001.zip › ijerph-1507237-supplementary.pdf]

SURVEY QUESTIONNAIRE

Student Science Club

Department of Oncology and Radiotherapy

Medical University of Silesia in Katowice

Dear Participants,

Number of cancer patients, who use some unconventional treatments is still growing. We would like to study this subject.
The aims of this study were to investigate complementary and alternative medicine (CAM) use amongst patients, access main sources of information about CAM and patient's opinion about effects of complementary and alternative treatment methods they used.

Complementary and alternative medicine is not conventional treatment. Traditional therapies were for example: chemotherapy, radiotherapy, hormone therapy and steroid therapy.

The questionnaire is anonymous. Please answer all questions.

Date ……………………………………………..

Gender

- ☐ female
- ☐ male

Age ……………………………………………….

Education

- ☐ primary
- ☐ vocational
- ☐ secondary
- ☐ higher

Profession…………………………………………..

Place of residence

- ☐ village
- ☐ city <50 thous. inhabitants
- ☐ city 50 - 200 thous. inhabitants
- ☐ city > 200 thous. Inhabitants

Voivodeship………………………………………..

1. Have you ever heard about CAM?
   - ☐ Yes
   - ☐ No
   - ☐ Difficult to claim

<u>If you indicated “no” or “difficult to claim” you should stop the questionnaire and go to last page.</u>

2. Did you consider to use CAM?
   - ☐ Yes
   - ☐ No
   - ☐ Difficult to claim

3. Have you ever used alternative methods?

- ☐ Yes
- ☐ No
- ☐ Difficult to claim

<u>If you indicated “no” you should go to question 16.</u>

4. What kind of CAM modalities did you use as alternative medicine?
   - ☐ high doses of vitamin C
   - ☐ herbs
   - ☐ special diets
   - ☐ amygdalin
   - ☐ hyperthermia
   - ☐ antineoplastons
   - ☐ hydrogen peroxide
   - ☐ baking soda
   - ☐ osteopathy
   - ☐ shark cartilage
   - ☐ homeopathy
   - ☐ marijuana
   - ☐ bioresonance therapy
   - ☐ NIA method
   - ☐ acupuncture
   - ☐ other

5. What sources of information about alternative methods of cancer treatment did you get? (*multiple choice*)
   - ☐ press
   - ☐ internet
   - ☐ tv
   - ☐ family
   - ☐ friends
   - ☐ other patients
   - ☐ physicians and medical workers
   - ☐ informative brochures

6. What form alternative methods of treatment did you use?
   - ☐ as the only treatment instead of traditional treatment
   - ☐ as the supportive therapy of traditional cancer treatment
   - ☐ as the therapy after completion of the proposed treatment
   - ☐ other …………………………

7. Did you inform your oncologist about used alternative methods?
   - ☐ Yes
   - ☐ No
   - ☐ Difficult to claim

8. What expectations do you have using alternative methods of cancer treatment? *(multiple choice)*
    - complete recovery
    - increasing effectiveness of conventional cancer treatment
    - no precise expectations, using every possibilities of treatment
    - other………………………..

9. When did you decide to use alternative therapy?
    - before traditional cancer treatment
    - during traditional cancer treatment
    - when traditional cancer treatment was completed or without expected results
    - other

10. Did you feel any positive effects of alternative therapy?
    - Yes
    - No
    - Difficult to claim

11. Did you feel any negative effects of alternative therapy?
    - Yes
    - No
    - Difficult to claim

12. Did you consider resigning from traditional cancer treatment for alternative therapy?
    - Yes
    - No
    - Difficult to claim

13. Would you recommend <u>used alternative methods</u> to other patients?
    - Yes
    - No
    - Difficult to claim

14. Have you still used alternative methods of cancer treatment?
    - Yes
    - No
    - Some of them yes, some of them no

<u>If you indicated "yes" you should go to question 16.</u>

15. What was the reason for resigning from alternative methods of cancer treatment? *(multiple choice)*
    - no positive effects
    - negative effects and / or complications
    - encouraged to resign by a doctor / other medical professional
    - encouraged to resign by family / friends
    - negative information about using method

- ☐ running out of funds to continue the therapy
- ☐ other……………………………..

16. From what sources did you know about CAM?
    (multiple choice)
    - ☐ I have never been interested in this topic
    - ☐ press
    - ☐ internet
    - ☐ tv
    - ☐ family
    - ☐ friends
    - ☐ physicians and medical workers
    - ☐ other patients
    - ☐ informative brochures

17. Did you talk with a doctor about possibility of using CAM?
    - ☐ Yes
    - ☐ No
    - ☐ Difficult to claim

18. What is your opinion about influence of CAM on health?
    - ☐ no influence
    - ☐ beneficial
    - ☐ harmful
    - ☐ difficult to claim

19. Should CAM be restricted by law?
    - ☐ Yes
    - ☐ No
    - ☐ Difficult to claim

20. Did you use complementary methods during conventional oncological treatment?
    - ☐ Yes (what?)……………………..
    - ☐ No
    - ☐ Difficult to claim

<u>If you indicated "no" or "difficult to claim" you should go to question 23.</u>

21. What was the reason of using complementary methods?
    - ☐ improve of well-being
    - ☐ improve of morphological and biochemical test parameters
    - ☐ strengthen immunological system
    - ☐ increase appetite / prevent weight loss
    - ☐ reduce the side effects of conventional oncological therapy

- ☐ other…………………

22. Did you notice some effects of using complementary therapy?
    - ☐ yes, only positive
    - ☐ yes, only negative
    - ☐ positive and negative
    - ☐ no
    - ☐ difficult to claim

23. Please indicate your satisfaction of conventional oncological treatment

0	1	2	3	4	5	6	7	8	9	10

very bad very good

24. Please indicate your satisfaction of relation with your oncologist

0	1	2	3	4	5	6	7	8	9	10

very bad very good

Cancer localization……………………………………………………

Date of diagnosis…………………………………………………….

Metastases

- ☐ Yes
- ☐ No
- ☐ I do not know

Intention of planned treatment

- ☐ curative
- ☐ palliative
- ☐ difficult to evaluate

Stage of disease

- ☐ early
- ☐ local and/or regional development
- ☐ metastatic
- ☐ difficult to evaluate
